# Supplementary figures and images for: Mapping potential connections between Southern Africa’s elephant populations
Source: PLoS One. 2022 Oct 11;17(10):e0275791. doi: 10.1371/journal.pone.0275791 (PMC9553058; doi:10.1371/journal.pone.0275791)

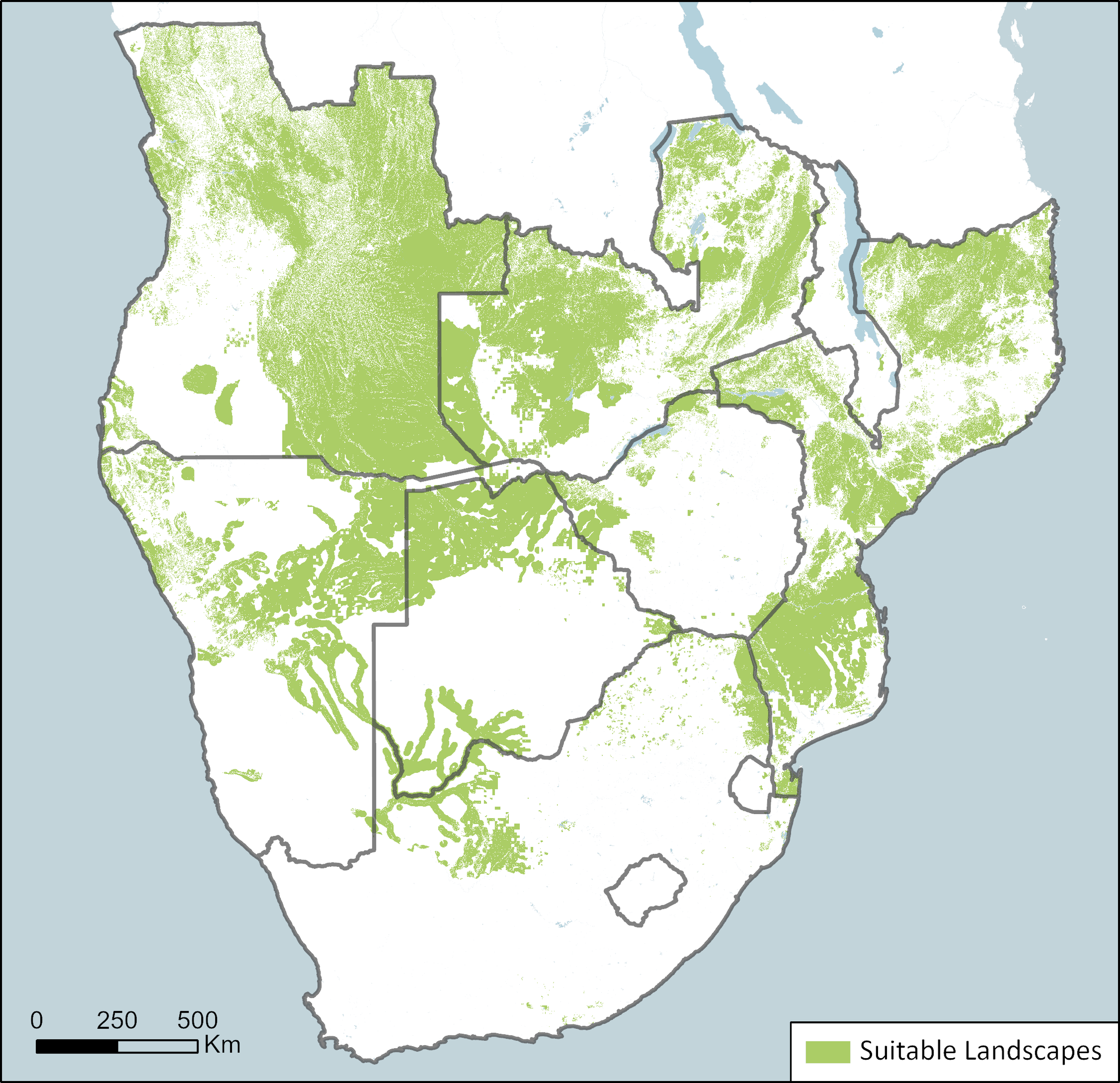

Supplement: S1 Fig — High resolution map showing areas that are both environmentally suitable for elephants and currently experience low human activity. (TIF) [file pone.0275791.s003.tif]

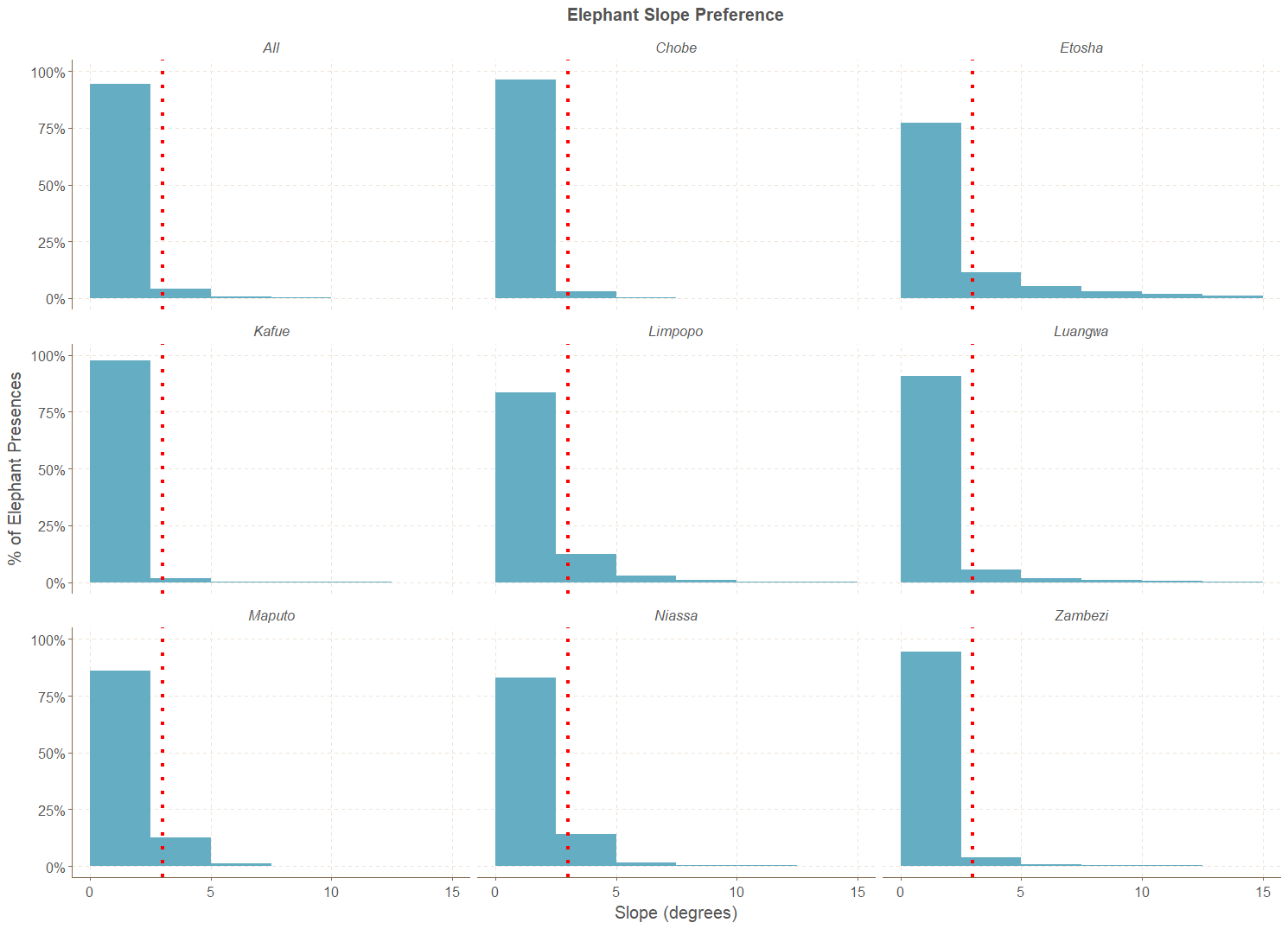

Supplement: S2 Fig — Histogram of elephant telemetry points at various slopes for each metapopulation cluster. The red dashed line indicates the threshold (3°) of preference for suitability. (TIF) [file pone.0275791.s004.tif]

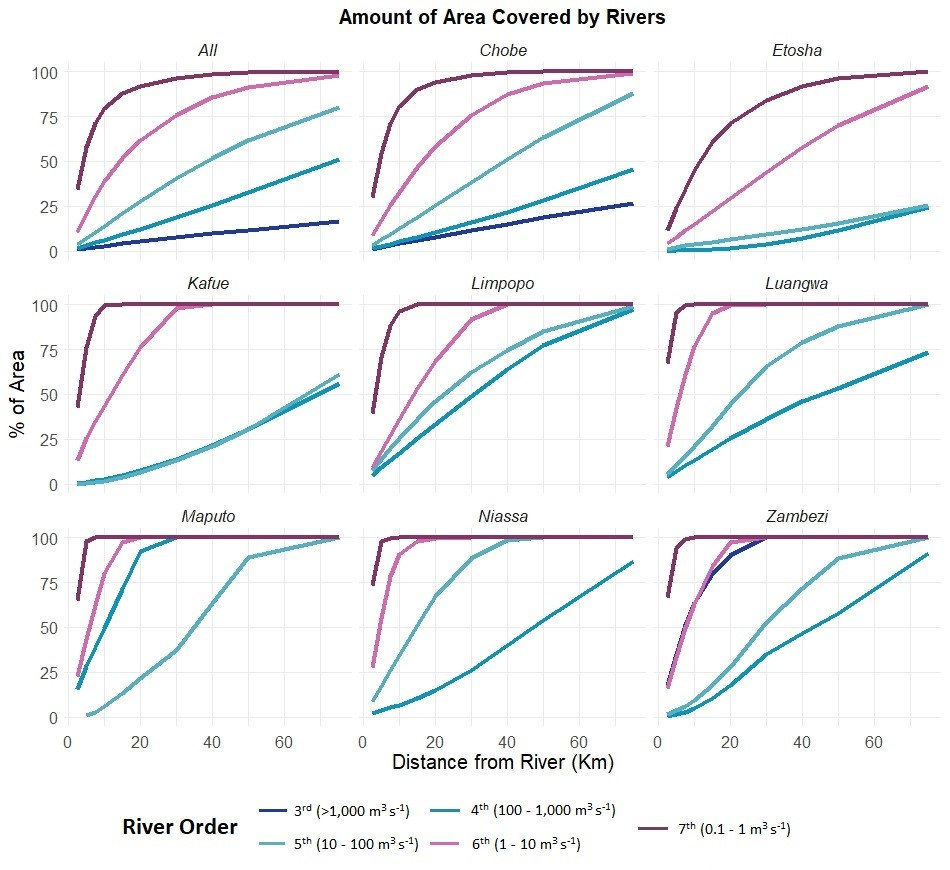

Supplement: S3 Fig — Accumulation curve of area within a conservation cluster as the distance increases away from rivers of varying flow orders. (TIF) [file pone.0275791.s005.tif]

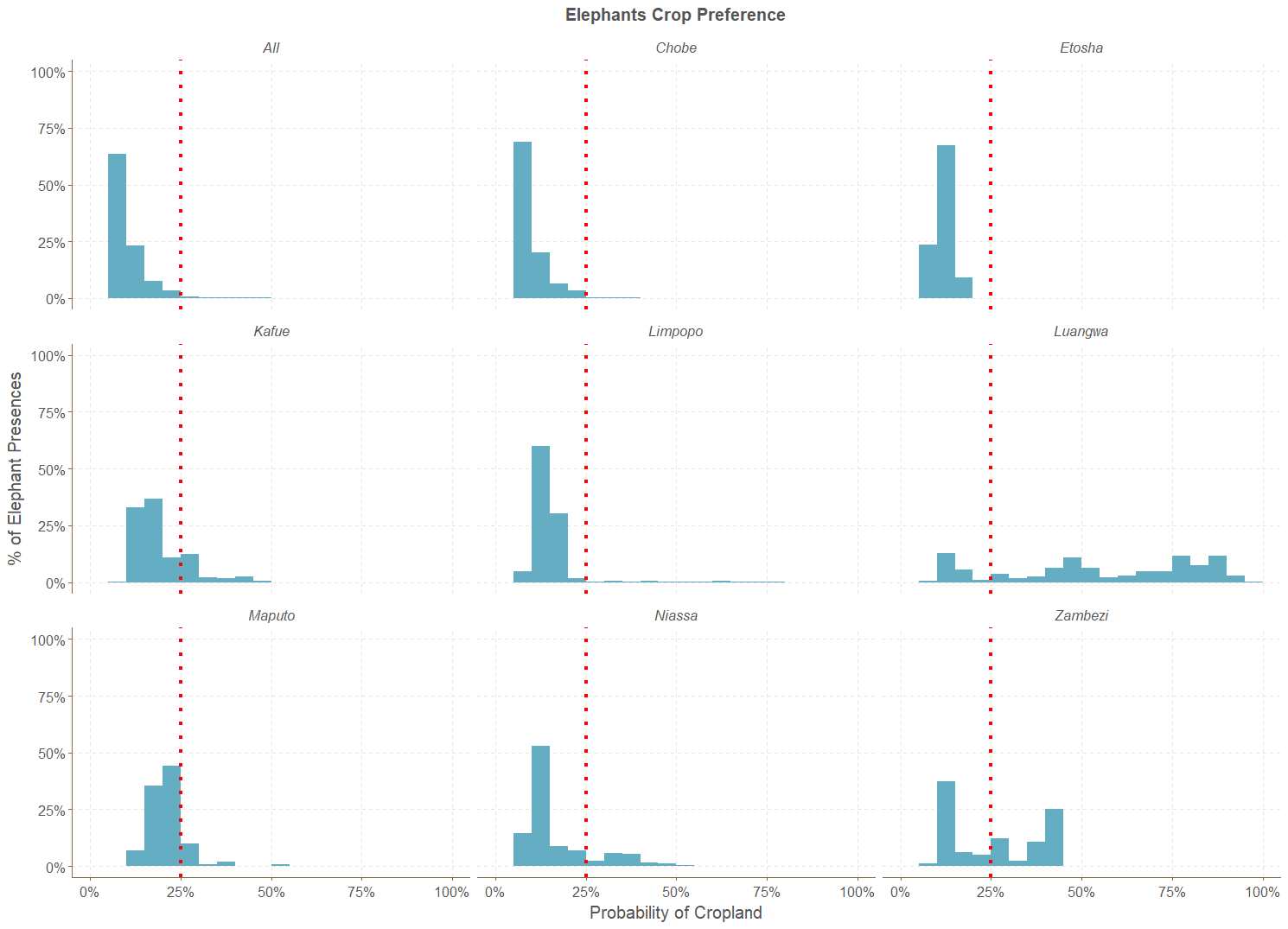

Supplement: S4 Fig — Histogram of elephant telemetry points at various cropland probabilities for each metapopulation cluster outside of protected areas. The red dashed line indicates the threshold (25%) of preference for suitability. (TIF) [file pone.0275791.s006.tif]

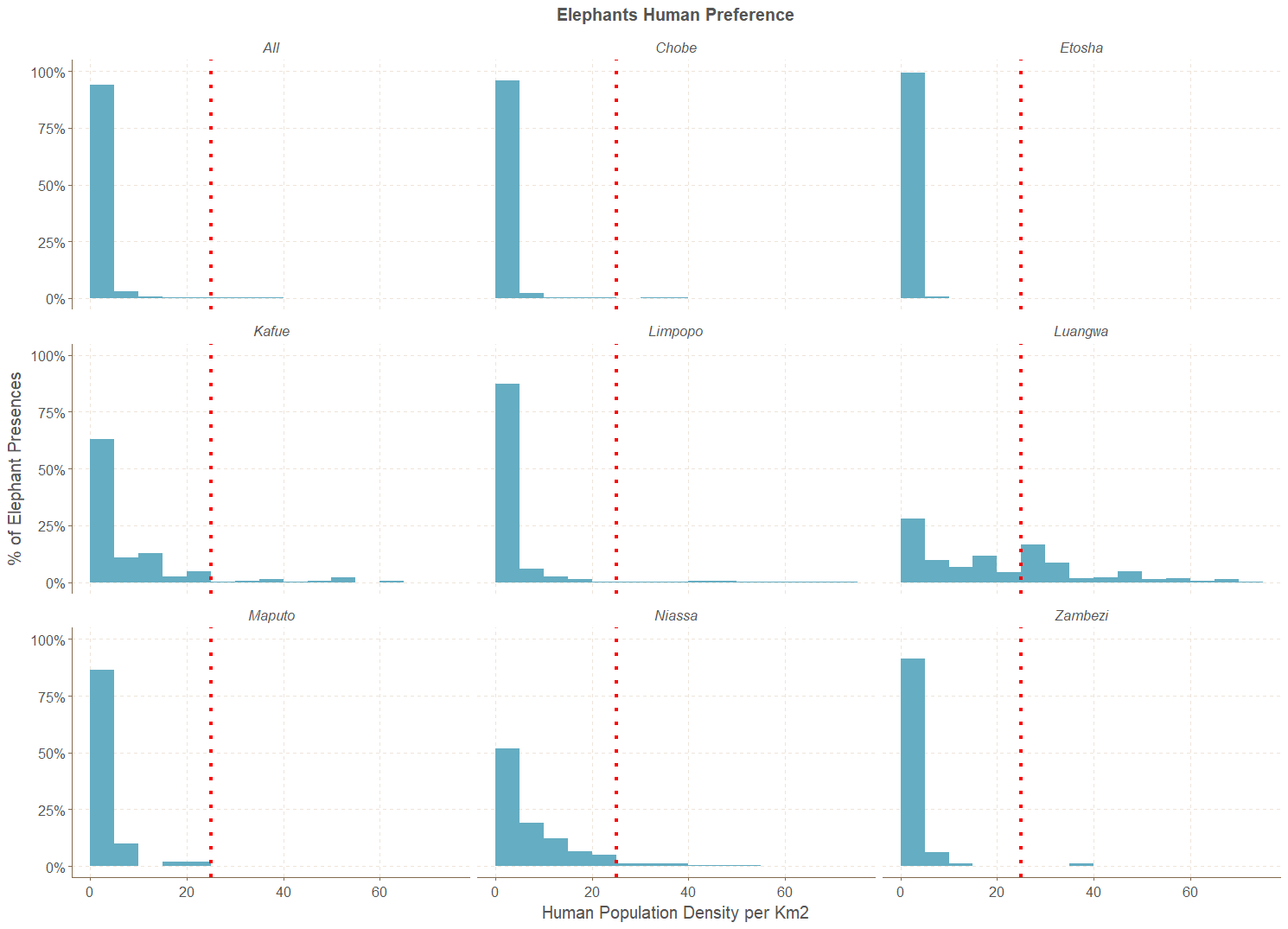

Supplement: S5 Fig — Histogram of elephant telemetry points at various human population densities for each metapopulation cluster outside of protected areas. The red dashed line indicates the threshold (25 people per km2) of preference for suitability. (TIF) [file pone.0275791.s007.tif]

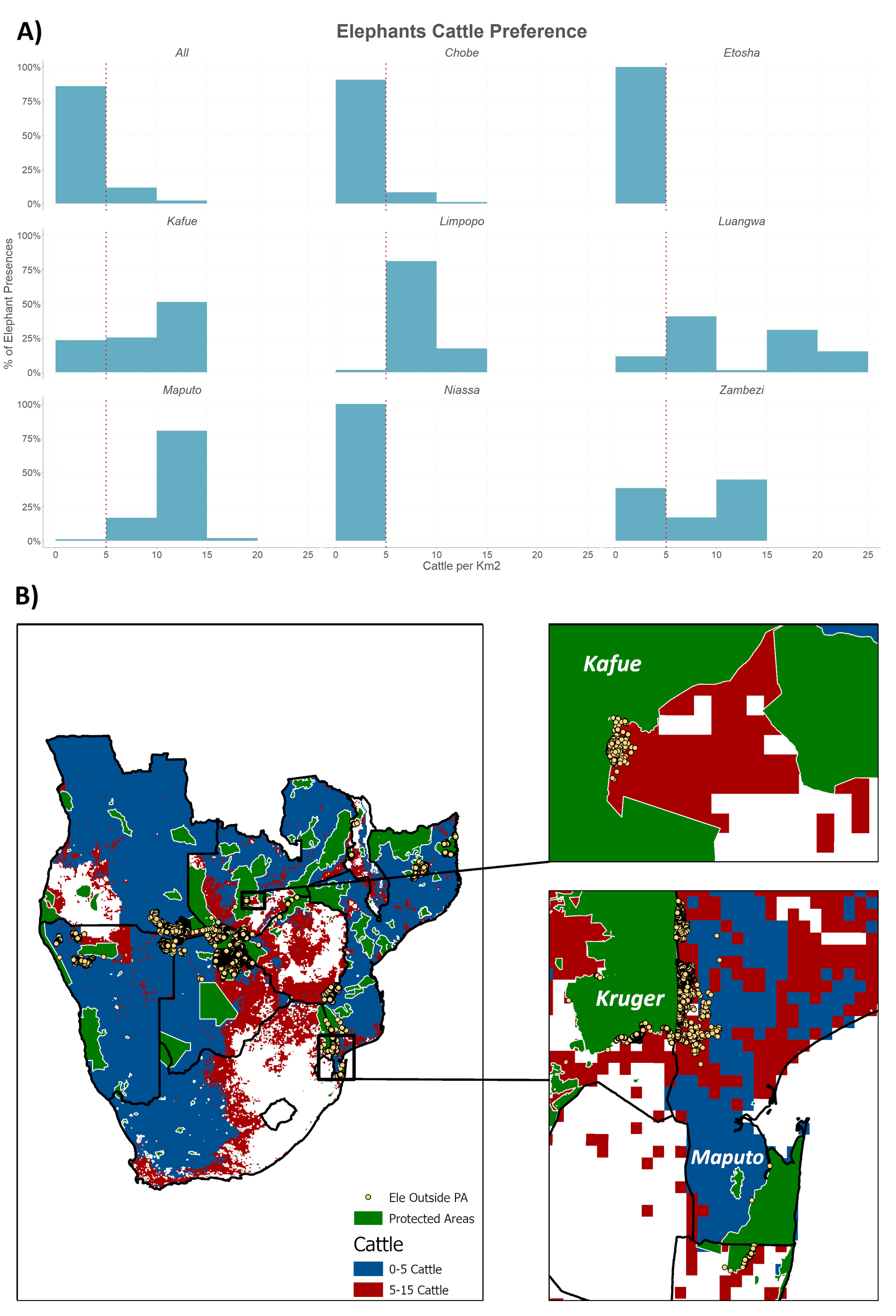

Supplement: S6 Fig — A) Histogram of elephant telemetry points at various cattle densities for each metapopulation cluster outside of protected areas. The red dashed line indicates the threshold (5 cattle per km2) of preference for suitability. B) A map of elephant telemetry points illustrating how spill over from protected areas leads to interactions with areas of high cattle density. (TIF) [file pone.0275791.s008.tif]
