# Supplementary material for: Mapping potential connections between Southern Africa’s elephant populations
Source: PLoS One. 2022 Oct 11;17(10):e0275791. doi: 10.1371/journal.pone.0275791 (PMC9553058; doi:10.1371/journal.pone.0275791)
Supplement: Attachment — Submitted filename: PONE-D-22-17386 Comments to Authors.docx [file pone.0275791.s009.docx]

PONE-D-22-17386: **Defragmenting Southern Africa’s elephant populations**

One of the main strengths of this work lies in the amount of telemetry and environmental/anthropogenic data collected over space and time by the authors, although as they are presented in the methodology, they do not explicitly indicate which research questions/objective they intend to answer with these data. For example, lines 97-106 are describing data acquisition from various sources but which objectives (which should have been clearly stated in the introduction section) are they addressing?

The statement in the “lines 110, 143, 164&254” for example, should have been used to guide the formulation and write up of this manuscript-understanding suitable habitats for elephants in southern Africa and how artificial barriers from different areas are limiting their connectivity and how to respond to these barriers/challenges, use lessons from these to set best practices that are context based?-unfortunately the paper as it stands, it does not reflect that this is the case.

One major flaw of the paper is the inconsistencies in writing and presentation of information-in the course of reading this, it was difficult to follow clearly from the title, introduction, methods, what exactly authors were trying to address….for example, one would ask, **what is the objective of the paper?** Is it about what the titles states? If so, one would have expected authors to set the scene and the aim of the paper, right from the introduction, however, this seems rather missing.

**Reservations in the specific sections of the paper**

**Title**

Despite the being rather ambitious, it would rather have some more action words on how realistic this can be given the current rate of local and global changes, that include among others climate and human population change and associated pressures

**Abstract**

The abstract seems a little twisted into a more descriptive than a conventional scientific way of presenting a research work with clear aim, methods results and conclusion…

**Introduction**

It would be good to see this section explaining explicitly the clear goals of the paper so readers can follow how this resonates with the subsequent sections. Also, authors could mention and describe sooner in the manuscript a sentence about the elephants populations being presented in this paper and cite the relevant figure (e.g. Fig.2) as well as a mention of the major threats that each of this is facing and the way defragmenting process would help address/mitigate these threats

Lines 80-84: It would be good to state if these hypothesis and based on which assumptions? And how are they linked with the objectives of the paper?

**Methods’ section**
Being a research article, it would be better to state sampling (data collection) strategy, sample size (e.g. source and number of individual elephants collared in each population, number of protected areas involved in data collection), data collection and analysis (including those other than collaring data) separately, rather than the present arrangement in the methodology section. For example, one would want to know, which sections in the “methods-lines 85-364” are methods and which ones are results? And what do all case studies in this section represent?-they are not stated anywhere in the introduction section and in the methodology it is not clear why they are being presented.

Which research question/objective do long-term telemetry data (lines 86-96) intend to answer in this paper? Is this backed up by any background hypothesis in the introduction section of the paper? These should have come a bit earlier in the introduction section of the paper so they guide the subsequent sections. For example, if questions in lines 110, 143&254 came out clearly in the introduction section, then the readers would be able to follow easily the flow of the story of this paper. Without this, it is difficult to have a meaningful interpretation and discussion of the results

What is SRTM in line 99?

There is no clear link between the subsequent sections of the paper (after introduction section) and the title of this paper. For example, not sure how one would expect elephants to cross fenced areas and how this would be resolved by telemetry data to achieve the ultimate goal which is reconnecting the populations.

I have looked at the manuscript and found a number of issues and problems related to the scientific clarity. Clearly the paper doesn't seem to state clear objective and rationale, and the methodology is not well formulated and implement in accordance to the title of the paper which makes interpretation of the synthesized information difficult to readers.

In view of the comments above, I am afraid that I cannot recommend this manuscript for publication in PLOS ONE.
